# Could work-related muscle activity explain sex differences in neck pain? A meta-analysis of a pooled dataset

**DOI:** 10.5271/sjweh.4227

**Published:** 2025-07-01

**Authors:** Markus Koch, Lars-Kristian Lunde, Mikael Forsman, Lars Louis Andersen, Markus Due Jakobsen, Mikkel Brandt, Henrik Enquist, Gisela Sjøgaard, Karen Søgaard, Xuelong Fan, Kaj Bo Veiersted

**Affiliations:** 1 National Institute of Occupational Health, Research group for Work Psychology and Physiology, Oslo, Norway; 2 Karolinska Institute, Department of Public Health Sciences, Division of Occupational and Environmental Medicine, Stockholm, Sweden; 3 KTH Royal Institute of Technology, School of Engineering Sciences in Chemistry, Biotechnology and Health Division of Ergonomics, Huddinge, Sweden; 4 National Research Centre for the Working Environment, Musculoskeletal Disorders and Physical Workload, Copenhagen, Denmark; 5 Lund University, Division of Occupational and Environmental Medicine, Lund, Sweden; 6 University of Southern Denmark Department of Sports Science and Clinical Biomechanics, Odense, Denmark; 7 University of Southern Denmark, Department of Clinical Research, Odense, Denmark; 8 Uppsala University, Department of Medical Sciences; Occupational and Environmental Medicine, Uppsala, Sweden

**Keywords:** musculoskeletal pain, occupational health, surface electromyography, upper trapezius, work break

## Abstract

**Objectives:**

Sustained activity of the upper trapezius muscle during work has been linked to the development of neck pain. Women have higher occurrences of neck pain than men, even in the same occupations. This study aimed to investigate sex-specific associations between upper trapezius muscle activity time-related variables and neck pain using a meta-analysis of pooled data.

**Methods:**

Seven Scandinavian research institutes provided surface electromyographic (EMG) data on the upper trapezius muscle activity during work and related questionnaire-based data on neck pain severity. EMG and questionnaire data were harmonized and pooled. Associations between upper trapezius muscle activity variables [median muscle activity, frequency of muscular rest periods, and periods with sustained muscle activity (SUMA)] and neck pain severity were investigated separately for women (N=293) and men (N=418) using linear regression analyses.

**Results:**

In the cross-sectional analyses, women showed significant positive associations between the number of short SUMA periods and negative associations for long SUMA periods in regard to neck pain severity. In the longitudinal analyses, women showed no significant associations. In the cross-sectional analysis for men, one significant positive association was found between median upper trapezius muscle activity and neck pain severity.

**Conclusions:**

Compared to men, neck pain severity among women appears to be more dependent on upper trapezius muscle activity patterns at work. Therefore, ergonomic and organizational recommendations for work should be sex-specific or adjusted for women to reduce their prevalence of neck pain. Further research is needed to elucidate the underlying mechanisms of these sex differences.

Neck pain is common in the working population, and women report it more frequently than men (1). Specifically, work involving arms above shoulder height, a forward-bent trunk, and precision tasks (2), as well as psychological and social factors (3) have been identified as possible risk factors for neck pain. A previous review found strong evidence that awkward or elevated arm posture poses a greater risk for women than men, but there was no conclusive evidence of sex-specific differences in the risk of neck-shoulder complaints associated with job demands, job control, or social support (4).

While some sex differences may be due to different tasks performed despite similar job titles (5), one possible explanation for the higher prevalence of neck pain among women may be linked to physiological differences (6). For example, women on average have lower muscle strength (7), which requires greater relative muscle activation to handle the same external forces as men (8). A higher relative load may increase the metabolic load (9), with changes reaching the level of nociceptor activation causing pain and fatigue (10). However, even the same relative load may have different effects on muscle metabolism due to hormonal differences (11). Differences in pain thresholds between women and men have also been hypothesized (12). Additionally, smaller body anthropometry may increase the movements performed in awkward postures or extreme joint positions. As a result, women may use different movement strategies or activation patterns when lifting (7).

Numerous studies have investigated the relationship between neck pain, muscular load, and activation of the upper trapezius muscle. Higher activity in the upper trapezius muscle among women has been found during manual working tasks requiring the arms to be lifted (13), during shoulder flexion (14), and during repetitive industrial work (15). If the higher mean/median upper trapezius muscle activity observed in these studies could be a possible cause of neck pain, significant positive associations should also be found. However, conflicting results from previous studies suggest that there is limited evidence for an association between increased mean upper trapezius muscle activity and the occurrence of neck pain (16–18). Another approach to studying the association between muscle activity and neck pain is to look at periods of sustained muscle activity (SUMA) >0.5% of the muscle activity achieved during an individual maximal voluntary isometric contraction (MVIC). This approach focuses on periods where type 1 muscle fibers are already active and will be exhausted over time (Cinderella hypothesis) (19). Periods of muscle activity below this threshold allow the muscles to recover, since almost all motor units are inactivated and no force is generated (20, 21). A few studies on this topic have shown significant negative associations between short SUMA periods <20 seconds and neck pain severity (22), and significant positive associations between long SUMA periods [>10 minutes (22), and >8 minutes (23)] and neck pain severity; however, these studies only included male participants. Hanvold and colleagues (24) found positive associations when measuring the total duration of SUMA with neck pain severity and duration in both sexes of young adult workers. In stratified longitudinal analyses, the results remained significant only among men. Previous unstratified analyses of the data used in this article found positive cross-sectional associations between short SUMA periods and neck pain and negative associations between long SUMA periods and neck pain severity (25).

With only these few studies available, the results on sex-specific differences in the associations between SUMA in the upper trapezius muscle and neck pain are still limited. Except for the preliminary study (25), the aforementioned studies included only a few specific occupations (harvesters, forwarders, researchers, hairdressers, electricians) with a small number of subjects per group. To overcome this limitation, further studies with a larger and more diverse group of participants, including individuals of both sexes and various occupations, are needed.

This study aimed to investigate sex-specific associations between upper trapezius muscle activity time-related variables (frequency of muscular rest periods and SUMA periods) and the occurrence of neck pain in a pooled data set from seven Scandinavian research institutes.

## Methods

Seven Scandinavian research institutes provided surface electromyography (EMG) data for upper trapezius muscle activity during working hours and corresponding questionnaire data on personal information and the severity of neck pain collected from 748 participants. All data were previously used in other studies (23, 24, 26–40). For this study, the different data sets were merged into a single database. The final database was checked for quality and completeness, ie, both EMG data and questionnaires were available for each participant.

### Study population

The final dataset included 711 participants (418 men, 293 women) with one workday’s EMG data and corresponding cross-sectional neck pain measures. Additionally, longitudinal neck pain information measured 6, 12 or 24 months after baseline was available for 259 of these participants (143 men, 116 women).

Sex information was collected by questionnaire, and we assumed that the participants responded based on their biological sex. In total, data were collected from 31 different occupations. A detailed overview of the composition of each occupation for the later analyses can be found in the supplementary material (www.sjweh.fi/article/4227), table S1).

### Objective measures – EMG

Upper trapezius activity was measured by EMG in all the included studies following the standards for sensor placement, measurement, and data processing (41, 42). The EMG measurement specifications (electrode placement, measurement frequency, and procedure for MVIC) for the different datasets can be found in the protocol article for this study (43). The EMG data were quality controlled, transformed into a standardized root-mean-square (RMS; eight values per second) format, and normalized to the MVIC. EMG data deviating from the RMS frequency of 8 Hz were interpolated to 8 Hz using spline interpolation. All data were noise-corrected by subtracting the noise level from all samples. The noise level was determined as the minimum of a moving average over 19 samples, from the corresponding recording (17, 44, 45).

The following variables were calculated bilaterally: the relative resting time (RRT), which represents the percentage of the total work duration with very low muscle activity <0.5% MVIC (46); the number of gap periods per minute with very low muscle activity <0.5% MVIC, representing the short episodes of “muscle rest” with a minimal duration of 0.125 second (17); and the median of muscle activity during the working day (45). In addition, the number of periods per hour of SUMA >0.5% MVIC with different lengths of continuous activity ranging from 1.5– >20 minutes was analyzed (22).

### Subjective measures – neck pain

In all included studies, information on the severity of neck pain was collected retrospectively by questionnaire, with the time period varying between the previous 24 hours, the previous 7 days, and the previous 4 weeks. Neck pain severity was assessed on a visual analog scale (VAS) and a 4-point scale (response categories: “not troubled”, “slightly troubled”, “somewhat troubled”, “severely troubled”). A detailed overview of the specific questions and their rating scales can be found in the protocol article (43). All the different measures of neck pain severity were combined into a common data set (43). We converted the scores of the 4-point scale to VAS scores in order to include all participants and maintain the accuracy of the VAS ratings.

For cross-sectional analyses, EMG and neck pain measures collected simultaneously at baseline were included, whereas for longitudinal analyses, the time for the questionnaire collection ranged from 6 to 24 months following the EMG measurements.

### Statistical analyses

We used the independent two-sample t-test to assess group differences between male and female participants. We performed linear regression analyses to examine associations between individual factors [age, height, weight, body mass index (BMI), smoking status] as predictor variables and the cross-sectional and longitudinal neck pain measures as outcome variables.

We used all EMG parameters separately as predictors in linear regression analyses to assess their association with cross-sectional and longitudinal neck pain severity (see supplementary table S2). We included individual factors significantly associated with neck pain severity as confounders in subsequent linear regression analyses between EMG parameters and neck pain severity. Specifically, for men, we adjusted longitudinal analyses of neck pain severity for age. No confounders were included in the analyses for women.

We calculated the 33^rd^ and 66^th^ percentiles of the total study population using the average muscle activity of the right upper trapezius. We then used these values to group participants according to the intensity of their physical workload and used the Pearson chi-squared test to determine whether the gender distribution within these groups differed.

For this study, regression analyses were performed separately for each sex. See previous research for analyses of the total sample, which included both men and women (25). Due to the large number of analyses, the false discovery rate (FDR) for the regression analyses between EMG parameters and neck pain and the comparison of EMG parameters between the sexes was controlled using the procedure described by Benjamini & Hochberg (47). Statistical analyses were performed using IBM SPSS Statistics 25 (IBM, Armonk, New York, USA).

## Results

The participants had a mean age of 39.8 [standard deviation (SD) 12.1] years, weight of 76.4 (SD 14.9) kg, height of 174.2 (SD 9.4) cm, and BMI of 25.0 (SD 3.6) kg/m^2^. The men were significantly younger, taller, and heavier than the women. The women reported significantly higher neck pain in both cross-sectional and longitudinal analyses (table 1).

**Table 1:** Descriptive statistics of the study sample and pain measurements (N=711). Sex differences were tested using a two-sample t-test.[SD=standard deviation; VAS=visual analog scale.]

		Total		Women		Men	P-value
		Mean (SD)		Mean (SD)		Mean (SD)	
Age (years)		39.8 (12.1)		41.1 (12.0)		38.9 (12.1)	<0.05
Weight (kg)		76.4 (14.9)		66.0 (10.2)		83.2 (13.5)	<0.05
Height (cm)		174.2 (9.4)		166.1 (6.2)		179.7 (6.8)	<0.05
BMI (kg/m^2^)		25.0 (3.6)		23.9 (3.2)		25.7 (3.7)	<0.05
Intensity of neck pain (VAS)							
	Cross-sectional	2.2 (2.5)		2.6 (2.8)		1.8 (2.2)	<0.05
	Longitudinal	1.9 (2.0)		2.1 (2.2)		1.6 (1.9)	<0.05

Regarding the activity of the upper trapezius muscle during work, the women had a significantly lower number of gaps per minute, fewer short SUMA periods (1.5–5, 5–10, 10–20, and 20–60 seconds) and more SUMA periods >20 minutes on both sides of the body (see table 2). In addition, the women had more SUMA periods of 10–20 minutes in the right upper trapezius muscle. No significant differences were found between men and women in the distribution of participants between workload intensity groups (see supplementary table S3).

**Table 2:** Electromyographic (EMG) parameters of left and right trapezius muscle for both sexes (N=711). P-values were controlled for false discovery rate. Sex differences were tested using a two-sample t-test. Gaps are expressed as the number of short breaks per minute. Sustained muscle activity (SUMA) is described by the number of periods per hour with muscle activity >0.5% MVIC. [MVIC=maximal voluntary isometric contraction; RRT=relative resting time; SD=standard deviation].

		Left upper trapezius			P-value	Right upper trapezius			P-value
		Women		Men		Women		Men	
		Mean (SD)		Mean (SD)		Mean (SD)		Mean (SD)	
RRT (% time)		17.9 (14.1)		17.7 (15.7)		16.4 (13.0)		15.5 (14.2)	
Gaps (N/minute)		6.0 (5.0)		8.2 (7.1)	<0.001	4.5 (3.2)		6.3 (5.2)	<0.001
Median (% MVIC)		3.6 (2.6)		3.2 (2.4)		4.3 (3.3)		4.1 (3.1)	
SUMA – periods (N/hour)									
	1.5–5 seconds	490.3 (344.1)		683.6 (472.4)	<0.001	514.3 (358.8)		676.9 (467.0)	<0.001
	5–10 seconds	187.2 (123.7)		250.3 (162.6)	<0.001	205.8 (139.2)		253.2 (163.4)	<0.001
	10–20 seconds	135.9 (79.9)		170.4 (100.2)	<0.001	150.9 (91.8)		177.9 (102.3)	<0.01
	20–60 seconds	134.6 (66.3)		154.4 (77.2)	<0.05	146.5 (72.2)		159.7 (78.2)	
	1–2 minutes	42.4 (21.4)		41.0 (24.2)		44.1 (22.7)		43.6 (24.5)	
	2–4 minutes	20.5 (12.7)		18.5 (14.5)		19.2 (12.5)		18.1 (13.8)	
	4–8 minutes	8.0 (7.3)		6.8 (8.4)		7.2 (6.9)		6.8 (7.8)	
	8–10 minutes	1.0 (1.5)		1.0 (1.9)		1.1 (2.0)		0.9 (1.6)	
	10–20 minutes	1.7 (3.0)		1.1 (2.5)	< 0.05	1.9 (3.3)		1.3 (2.6)	<0.05
	>20 minutes	0.8 (1.8)		0.3 (1.2)	<0.01	0.8 (2.0)		0.4 (1.4)	<0.05

### Associations between muscle activity and neck pain among women

*Cross-sectional analyses.* In the cross-sectional analyses, no significant associations were found between RRT, the number of gaps per minute, median EMG activity, and neck pain severity. Significant positive associations were found between short SUMA periods of 5–10 seconds (β 0.161, R^2^ 0.026, P<0.05), 10–20 seconds (β 0.191, R^2^ 0.036, P<0.01), 20–60 seconds (β 0.166, R^2^ 0.27, P<0.05), and neck pain severity on the left side of the body (see table 3). Also on the right side of the body significant positive associations were found for short SUMA periods of 1.5–5 seconds (β 0.152, R^2^ 0.023, P<0.05), 5–10 seconds (β 0.152, R^2^ 0.023, P<0.05), 10–20 seconds (β 0.167, R^2^ 0.028, P<0.05), 20–60 seconds (β 0.174, R^2^ 0.03, P<0.05) and neck pain severity. Associations of SUMA periods >2 minutes (left side of the body) and >4 minutes (right side of the body) with neck pain severity were negative. On the left side, a significant negative association was found between SUMA periods >20 minutes and neck pain severity (β -0.172, R^2^ 0.03, P<0.05). Similarly, on the right side, a significant negative association was found between SUMA periods 10–20 minutes and neck pain severity (β -0.217, R^2^ 0.047, P<0.01).

**Table 3:** Regression analysis scores (b-values) between electromyographic (EMG) variables and neck pain. P-values were corrected for false discovery rate. **Gray shading:** negative associations. **No shading:** positive associations. [RRT=relative resting time; SUMA=sustained muscle activity].

EMG variables		Cross-sectional neck pain								Longitudinal neck-pain						
		Women				Men				Women				Men*		
		β	R^2^	P-value		β	R^2^	P-value		β	R^2^	P-value		β	R^2^	P-value
Left																
	RRT	0.092	0.009			-0.061	0.004			-0.042	0.002			0.006	0.051	
	Gaps	-0.054	0.003			0.096	0.009			-0.127	0.016			0.065	0.055	
	Median	-0.034	0.001			0.048	0.002			0.014	0.000			0.010	0.051	
SUMA																
	1.5–5 seconds	0.128	0.016			0.078	0.006			-0.031	0.001			0.078	0.057	
	5–10 seconds	0.161	0.026	< 0.05		0.027	0.001			-0.036	0.001			0.050	0.053	
	10–20 seconds	0.191	0.036	< 0.01		0.018	0.000			-0.053	0.003			-0.002	0.051	
	20–60 seconds	0.166	0.027	< 0.05		-0.021	0.000			-0.087	0.008			-0.034	0.052	
	1–2 minutes	0.043	0.002			-0.076	0.006			-0.057	0.003			-0.052	0.053	
	2–4 minutes	-0.035	0.001			-0.018	0.000			0.090	0.008			-0.009	0.051	
	4–8 minutes	-0.072	0.005			0.040	0.002			0.114	0.013			0.069	0.055	
	8–10 minutes	-0.063	0.004			-0.006	0.000			0.041	0.002			0.061	0.054	
	10–20 minutes	-0.116	0.013			0.012	0.000			0.129	0.017			-0.030	0.051	
	>20 minutes	-0.172	0.030	< 0.05		0.040	0.002			-0.023	0.001			-0.042	0.052	
Right																
	RRT	0.037	0.001			-0.093	0.009			-0.039	0.001			-0.062	0.051	
	Gaps	0.075	0.006			0.044	0.002			-0.084	0.007			-0.024	0.047	
	Median	-0.026	0.001			0.143	0.020	< 0.05		-0.061	0.004			0.086	0.054	
SUMA																
	1.5–5 seconds	0.152	0.023	< 0.05		0.019	0.000			-0.068	0.005			-0.069	0.051	
	5–10 seconds	0.152	0.023	< 0.05		0.011	0.000			-0.045	0.002			-0.060	0.050	
	10–20 seconds	0.167	0.028	< 0.05		-0.002	0.000			-0.085	0.007			-0.046	0.049	
	20–60 seconds	0.174	0.030	< 0.05		-0.033	0.001			-0.074	0.005			0.007	0.047	
	1–2 minutes	0.105	0.011			-0.038	0.001			-0.040	0.002			0.079	0.053	
	2–4 minutes	0.027	0.001			0.000	0.000			-0.005	0.000			0.087	0.054	
	4–8 minutes	-0.088	0.008			0.076	0.006			-0.009	0.000			0.107	0.058	
	8–10 minutes	-0.063	0.004			-0.059	0.004			-0.033	0.001			-0.013	0.047	
	10–20 minutes	-0.217	0.047	<0.01		0.089	0.008			-0.088	0.008			0.050	0.049	
	>20 minutes	-0.093	0.009			0.053	0.003			0.135	0.018			-0.030	0.048	

* Regressions between EMG variables and longitudinal neck pain in men were adjusted for age

*Longitudinal analyses.* In the longitudinal analyses, no significant associations were found between upper trapezius muscle activity and neck pain severity among women.

### Associations between muscle activity and neck pain among men

Among men, only one significant positive association was found. This was between the median upper trapezius muscle activity of the right body side and neck pain severity (β 0.143, R^2^ 0.02, P<0.05) sectional analyses. Longitudinal analyses showed no significant associations. In addition, no clear pattern was found regarding the direction of possible associations between EMG parameters and neck pain severity (see table 3).

## Discussion

This study examined the sex-specific association between upper trapezius muscle activity patterns during work and neck pain severity across various occupations. In cross-sectional analyses, women with higher neck pain severity had more frequent short periods of SUMA and fewer longer periods of SUMA in the upper trapezius. Although not significant, it should be noted that many associations changed directions in the longitudinal analyses. For men, one significant association was found in the cross-sectional analyses: a positive relationship was determined between median upper trapezius muscle activity and neck pain severity.

### Neck pain severity

Consistent with previous research (1), women reported higher neck pain severity than men at both baseline and longitudinal measures. Both cross-sectional and longitudinal analyses included a variety of occupations for both sexes, reflecting a wide distribution of pain prevalence across occupations, consistent with previous research (48).

### Upper trapezius muscle activity

The present study found a lower number of gaps per minute, a lower number of shorter SUMA periods, and a higher number of longer SUMA periods among women compared to men. One can assume that women appear to have fewer opportunities to take breaks and recover during the workday. Previous studies have shown that women were less susceptible to fatigue than men in several muscle groups when isometric and dynamic shortening contractions were examined (49). These results from previous studies contradict the above-mentioned assumption that women “appear to have fewer opportunities”; on the contrary, they do not need as many breaks as men, and they are able to work for longer periods with sustained muscle activity in the upper trapezius. Considering muscle mass and strength differences, women might use different movement strategies than men, for example, when performing heavy work tasks. For instance, Jakobsen and colleagues (7) determined that women were found to have more short exposure periods in the vastus lateralis muscle, which means that they are more apt to use a type of leg strategy during manual lifting tasks in the workplace. However, regarding the upper trapezius muscle, they found that men had a higher number of short exposures in the upper trapezius muscle during the working day (7). When focusing on other strenuous work tasks, a higher number of longer SUMA periods in the upper trapezius were found among women. This might be explained by the fact that EMG measures can reflect both physical and psychosocial stress (50). Women may suffer from higher job strain (51) or a higher psychosocial strain during leisure time (52), which could increase the overall tension in the upper trapezius muscles.

### Associations between upper trapezius muscle activity and neck pain severity among women

In the cross-sectional analyses, women showed significant positive associations between short SUMA periods and neck pain severity but negative associations between long SUMA periods and neck pain severity. Women appear to adjust their upper trapezius muscle activity patterns in response to pain, ie, they need to take more frequent breaks and are unable to work for long periods of sustained activity to reduce or avoid pain (49). In the longitudinal analyses, no statistically significant associations were found. Based on a few significant findings from sub-analyses including only hairdressers (see supplementary table S4), it appears that working with short periods of muscle activity may be preventive in the long term, whereas working with long periods may be a risk factor for increased neck pain severity. For professions with specific work tasks and demanding body postures (eg, arms above shoulder height) like hairdressers, further studies on this topic are needed to confirm this hypothesis. The relationship between these adaptive muscle activity patterns and factors such as age or years of occupational exposure also provides an interesting opportunity for future research, as women may naturally start with longer SUMA periods that are subsequently modified as they develop neck pain during their working lives.

In a preventive context, the significant positive associations of SUMA periods of 10–20 minutes (right side of the body) and neck pain severity could indicate a feasible threshold for practical workplace interventions. A short break would be beneficial after continuous work with muscle strain of this duration. As a wide range of occupations were included, we assume that this threshold will need to be shifted to a shorter duration for heavier work tasks. A previous study of male harvesters, forwarders and researchers, whose data were included in our study, found a similar threshold of SUMA periods >8 minute for men (23). In addition, another of the included studies found a lower threshold (>4 minutes) for a group of young hairdressers, electricians, and students (24). Two important points merit discussion. First, for men, Østensvik et al (23) found negative correlations between SUMA periods <4 minutes, but positive correlations between SUMA periods >4 minutes in the upper trapezius and neck pain intensity in cross sectional analyses (23). Additionally, the authors found positive associations between SUMA periods >8 minutes and the probability of reporting pain >30 days a year in longitudinal analyses (23). To the contrary, our study indicated positive associations of short SUMA periods and negative associations of long SUMA periods with neck pain severity in cross-sectional analyses among women. This difference could be due to the specific and monotonous work tasks investigated by Østensvik et al. Assuming that their participants have been working in the same job for several years, it is possible that the cross-sectional findings could reflect longitudinal associations. Second, Hanvold et al (24) found a slightly lower threshold than we did. This difference could be due to the younger age group of their participants (21–25 years), who may not have fully adjusted to their occupational exposures. In addition, the occupations included in their study may be demanding on the neck, such as working with arms raised (hairdressers, electricians) or being exposed to both physical and psychosocial exposures (kindergarten workers).

### Associations between upper trapezius muscle activity and neck pain severity among men

For men, only a significant association between median EMG activity and neck pain severity was found in cross-sectional analyses. As mentioned above, significant associations for men were previously identified by Østensvik et al (23) and Hanvold et al (24) in two subsets of our data. Pooling these subsets with data from other occupations may have introduced a wider range of exposure when analyzing the associations with the different measures of neck pain, which may explain why our study did not find more significant results for men. The positive association between median EMG activity and neck pain severity in cross-sectional analyses shows that men with higher average activity in the upper trapezius muscle during the working day are more exposed to pain. Similar associations were found in a previous study that included both sexes when analyzing muscle activity during computer work (53). Another possible explanation for finding significant associations mainly among women is the impact of psychosocial factors on muscle activity, as previously mentioned. While role conflict is a risk factor and empowering leadership and decision control are protective factors for neck pain (3), women and men may differ in their exposure to these factors, depending on their type of work. An earlier review found only limited evidence of sex-specific differences in the associations between job demands, job control, social support, and neck pain (4). In contrast, a later study found that women were less exposed to job stress when they had higher levels of supervisory support, but this was not the case for men (51).

Additionally, men on average had lower neck pain severity, with a narrower gradient. This may have weakened the relationship between EMG parameters and neck pain severity. Choosing another body part where men report more pain than women, such as low-back pain, might have led to different results. The fact that men work less often with long periods of SUMA and more often with short protective periods of SUMA could possibly explain the lower severity rates among men.

### Practical implications

The findings of this study could potentially have practical implications for workplace ergonomics. Given the significant differences in muscle activity patterns and their associations with neck pain severity between men and women, ergonomic interventions should probably be tailored to each sex. For women in particular, implementing more frequent, shorter breaks and introducing task variation throughout the workday may help reduce the occurrence of prolonged SUMA periods and thereby possibly prevent neck pain. From a sports science perspective, preventive exercise could also help prevent neck pain by increasing muscle capacity. However, after a long and physically demanding day at work, this may not be an option for all workers. Regular assessment of muscle activity patterns and neck pain symptoms, particularly in high-risk occupations, may aid in early identification and prevention of neck pain issues. Implementation of these measures could potentially contribute to reducing the prevalence and severity of work-related neck pain, particularly among women. Nevertheless, more studies in this area are needed before final practical recommendations can be provided. These studies could also include other measurement systems, such as accelerometers or inertial measurement units, to examine movement patterns in the neck while simultaneously measuring upper trapezius muscle activity. This combination would make it possible to relate upper trapezius activity not only to pain, but also to movements during work tasks, and to distinguish upper trapezius activity from psychosocial influences.

### Strengths and limitations of the study

One major strength of this research is the number of participants included with high quality EMG data of the upper trapezius collected in the workplace and associated neck pain measures. To our knowledge, only one other study of this size (N=647) is comparable to ours (38). For both sexes, a wide range of occupations was included (see supplementary table S5). In addition, the intensity of the physical workload based on the median activity of the right upper trapezius muscle did not differ between the sexes (see supplementary table S3). Therefore, our results and the sex differences obtained can be interpreted as general and are not limited to individual occupational groups. Because the EMG measurements were taken during only one working day, they may not represent the full range of exposures in occupations with variable work tasks over days/weeks (eg, construction workers). For other occupations, such as hairdressers, a one-day measurement may be representative of the average exposure at work.

The pooling of several studies can also be seen as a limitation. The authors are aware that the combination of different neck pain measures (both different pain scales and temporal occurrence of pain) is debatable. To include as many participants as possible and maintain sensitivity in the pain scales, the authors collaboratively consented to the methodology described above. Similarly, pooling all the EMG data from the individual studies may introduce some bias into the study. As much of the data was recorded many years ago, we had to trust that the quality standards described were met. Based on the relevant publications, all data sampling followed the recommendations for surface electromyography for the non-invasive assessment of muscles [ SENIAM (41)], including sensor placement, reference measurements, and RMS calculation. All data were rechecked for quality after pooling.

Another limitation of the study is the number of analyses examined due to the large number of EMG variables. Using the Benjamini & Hochberg method (47), we tried to exclude false positive results. In general, no significant correlations were found in the longitudinal analyses in contrast to the cross-sectional analyses. This may be due to the smaller number of participants included. Unfortunately, not all studies that contributed data had longitudinal data available. Therefore, the occupations included differed between the cross-sectional and longitudinal analyses and may reflect a less informative context. If more participants had been included in the longitudinal analyses, more significant results might have been found due to statistical power.

Despite these limitations, the authors believe that the strengths of the study and the novelty of the findings represent a valuable contribution to the body of research on the causes of neck pain and possible sex-specific factors.

### Concluding remarks

Based on the results of this study, the severity of neck pain is more dependent on the muscular load of the upper trapezius muscle among women than among men. Therefore, we recommend that preventive ergonomic occupational health services should be tailored to the sexes. Women, because of their specific anatomical and functional characteristics, may particularly benefit from organizing work tasks and designing workplaces in a way that allows for more frequent rest. From a cost–benefit perspective for the employer, as well as from a health improvement perspective for the worker, preventive ergonomic exercises for the neck muscles could be beneficial for both sexes (54).

Regardless of sex, it is important to have regular breaks to prevent neck pain. Strengthening oneself or reducing the demands of tasks can help alleviate this issue for everyone. To clarify possible positive or negative effects of psychosocial factors on the associations between muscle activity in the upper trapezius muscle and neck pain, more studies are needed to examine both factors simultaneously.

## Supplementary material

Supplementary material

## Protection of research participants

All data is stored on a secure server at the National Institute of Occupational Health and is only available in anonymized format to the researchers.
