# Supplementary material for: Could work-related muscle activity explain sex differences in neck pain? A meta-analysis of a pooled dataset
Source: Scand J Work Environ Health. 2025 Jun 26;51(4):323–32. doi: 10.5271/sjweh.4227 (PMC12282473; doi:10.5271/sjweh.4227)
Supplement: Supplementary material [file SJWEH-51-323-S001.pdf]

Could work-related muscle activity explain sex differences in neck pain? A meta-analysis of a pooled dataset[1]

by Markus Koch, PhD,[2] Lars-Kristian Lunde, PhD, Mikael Forsman, PhD, Lars Louis Andersen, PhD, Markus Due Jakobsen, PhD, Mikkel Brandt, PhD, Henrik Enquist, PhD, Gisela Sjøgaard, PhD, Karen Søgaard, PhD, Xuelong Fan, PhD, Kaj Bo Veiersted, PhD

1. Supplementary material
2. Correspondence to: Markus Koch, National Institute of Occupational Health, Research group for Work Psychology and Physiology, Gydas vei 8, 0361 Oslo, Norway. [E-mail: Markus.Koch@stami.no]

Table S1. Number of participants included specified by occupations according to cross-sectional and longitudinal neck pain.

	**Cross-sectional neck pain**		**Longitudinal neck pain**	
	**Women**	**Men**	**Women**	**Men**
Occupation				
Assembly worker	9	16		
Assistant worker		3		1
Brewery worker	4			
Bricklayer		22		1
Carpenter		17		10
Cleaner	2		2	
Concrete worker		37		5
Cook or kitchen helper	4	4	2	
Electrician		16		11
Engineer	1	2	1	1
Firefighter		2		
Foreman		5		3
Gardener / forest worker	1	4		
Hairdresser	36		29	
Harvester / driver	2	92		84
Health care personal	70	6	33	2
Helicopter pilot / crew	1	17		
Machine operator	3	1		
Meat cutter	6	29		
Mechanic		4		
Office worker / secretary	79	28	25	18
Postal worker	1	35		
Project manager / leader	5	8	4	3
Retail personal	32	13	11	
Rubber mixing	7	1		
Student	5		4	
Surgeon	1	11		
Warehouse worker	9	29		
Windscreen inspection	7	3		
Working with various tasks	5	2	2	1
Other occupations	3	11	3	3
**Total**	**293**	**418**	**116**	**143**

Table S2. Regression analysis for individual factors on neck pain variables.

	Cross-sectional neck pain			Longitudinal neck pain		
	β	R^2	p	β	R^2	p
Women						
Age	0.098	0.010		0.058	0.003	
Weight	0.097	0.009		0.003	0.000	
Height	0.088	0.008		-0.030	0.001	
BMI	0.082	0.007		0.083	0.007	
Smoking	0.042	0.002		0.076	0.006	
Men						
Age	-0.006	0.000		0.216	0.047	< 0.01
Weight	0.021	0.000		0.101	0.010	
Height	-0.032	0.001		-0.003	0.000	
BMI	0.043	0.002		0.119	0.014	
Smoking	0.011	0.000		0.052	0.003	

Table S3. Distribution of participants according to physical workload intensity categories. The median activity of the right upper trapezius muscle was used to calculate the 33rd and 66th percentile across participants. Participants with a median activity below the 33rd were assigned to the light intensity group, participants with a median activity between the 33rd percentile and 66th percentile were assigned to the medium intensity group, and participants with a median activity above the 66th percentile were assigned to the high intensity group. *The sex distribution in the different physical workload intensity groups was not significantly different (p = 0.346; tested by Pearson's chi-square test).

	Physical workload			
**Distribution by occupation**	**Light**	**Medium**	**High**	**Total**
Assembly worker	2	10	13	25
Assistant worker	1	1	1	3
Brewery worker	0	1	3	4
Bricklayer	12	7	3	22
Carpenter	5	8	4	17
Cleaner	0	0	2	2
Concrete worker	15	13	9	37
Cook or kitchen helper	2	4	2	8
Electrician	2	8	6	16
Engineer	1	2	0	3
Firefighter	1	0	1	2
Foreman	3	2	0	5
Gardener / forest worker	1	0	4	5
Hairdresser	6	10	20	36
Harvester / driver	43	33	18	94
Health care personal	23	27	26	76
Helicopter pilot / crew	14	2	2	18
Machine operator	2	1	1	4
Meat cutter	6	16	13	35
Mechanic	3	0	1	4
Office worker / secretary	53	32	22	107
Postal worker	5	7	24	36
Project manager / leader	3	5	5	13
Retail personal	7	20	18	45
Rubber mixing	3	2	3	8
Student	4	0	1	5
Surgeon	1	1	10	12
Warehouse worker	9	12	17	38
Windscreen inspection	6	2	2	10
Working with various tasks	5	1	1	7

Other occupations	3	8	3	14
**Distribution by sex***				
Women	98	90	105	293
Men	143	145	130	418
**Total**	**241**	**235**	**235**	**711**

Table S4. Regression analysis scores (β-values) of a subanalysis between EMG variables and neck pain in female hairdressers. P-values were corrected for FDR. Gray shading: negative associations. No shading: positive associations. N = 36/29 (cross-sectional / longitudinal analyses).

	**Cross-sectional neck pain**			**Longitudinal neck pain**		
	β	R^2	p	β	R^2	p
**Left**						
RRT	0.176	0.230		-0.225	0.051	
Gaps	0.402	0.361	< 0.05	-0.139	0.019	
Median	-0.291	0.283		0.120	0.014	
SUMA						
1.5s - 5s	0.430	0.382	< 0.05	-0.110	0.012	
5s - 10s	0.411	0.364	< 0.05	-0.185	0.034	
10s - 20s	0.247	0.259		-0.280	0.079	
20s - 60s	0.092	0.207		-0.375	0.140	
1min - 2min	0.084	0.206		-0.288	0.083	
2min - 4min	-0.090	0.207		-0.073	0.005	
4min - 8min	-0.124	0.214		0.125	0.016	
8min - 10min	-0.120	0.213		-0.027	0.001	
10min - 20min	0.041	0.201		0.505	0.255	< 0.05
>20min	-0.247	0.260		0.140	0.020	
**Right**						
RRT	0.279	0.287		-0.188	0.035	
Gaps	0.324	0.314		-0.138	0.019	
Median	-0.429	0.390	< 0.05	0.051	0.003	
SUMA						
1.5s - 5s	0.407	0.373	< 0.05	-0.254	0.065	
5s - 10s	0.382	0.353	< 0.05	-0.291	0.084	
10s - 20s	0.332	0.318		-0.350	0.123	
20s - 60s	0.223	0.258		-0.404	0.163	
1min - 2min	0.162	0.235		-0.244	0.060	
2min - 4min	-0.008	0.210		0.080	0.006	
4min - 8min	-0.044	0.211		0.332	0.110	
8min - 10min	-0.092	0.218		0.236	0.056	
10min - 20min	-0.414	0.377	< 0.05	0.221	0.049	
>20min	-0.274	0.280		0.270	0.073	
